# The Future of Salisbury Infirmary

**Published:** 1920-09-18

**Authors:** 


### THE FUTURE OF SALISBURY INFIRMARY.

Considerable importance was given to the annual Court of Governors of Salisbury Infirmary by the presentation of the report of the Committee of Inquiry which was appointed last June to consider three points: 1. The building of an infirmary on a new site; 2. The rebuilding of the Infirmary on the present site; 3. The reconstruction of the present buildings. The advice of Mr. Silley, architect, was sought, because the alterations and extensions needed were extensive. He reported that it would not be prudent to attempt to alter or extend the existing buildings on the scale required, but that the committee should postpone important alterations until it had considered whether it would not be cheaper, in the end, and better for the patients and staff, to erect a new building on a suitable site which could be designed with a view to extension. The committee reported in favour of a new hospital on the outskirts of the town, to avoid the extravagance of reconstructing the present building, and pointed out that the local authorities are empowered to pay 75 per cent. towards the equipment and cost of maintenance of such new departments as those for maternity, school clinic, venereal disease and orthopædics in respect of cases which local authorities send to general hospitals. While no reliable figures can be obtained at present for building or reconstruction, the committee feels that the cost of a new hospital, however heavy, would be well repaid if it led to greater comfort for patients and more economical working. The committee is convinced that the public can be relied on to support an up-to-date hospital much more generously than an "old, extravagant building." Therefore, if a new hospital can be secured by spending half the present capital, together with the value of the present site, the committee thinks such expenditure is justified. The present site is condemned as crowded, low-lying and noisy.

Four sites in the neighbourhood were examined and preference given to that at Butt's Farm, in Castle Road, which comprises 68¼ acres and is for sale by the Ecclesiastical Commissioners for £5,950.

The sub-committee estimates the cost of a new hospital of 170 beds, at £1,740 per bed, at £304,000, including the site and redemption of tithe. The present value in the market of the infirmary's invested funds is £68,700, which provides an income of £4,108; consequently the sub-committee considers it out of the question to sacrifice capital at this rate. The value of the present infirmary buildings and grounds is £30,000. The sub-committee suggests that a large part of the new buildings should be huts if removal is thus decided on; and that large gifts from generous donors and grants from public authorities would be necessary to accomplish the transfer. The sub-committee therefore recommends the purchase of the Butt's Farm site, and a meeting be called to consider whether the the Inquiry Committee's recommendations can be carried out.

